# A Spatial Probit Econometric Model of Land Change: The Case of Infrastructure Development in Western Amazonia, Peru

**DOI:** 10.1371/journal.pone.0152058

**Published:** 2016-03-24

**Authors:** E. Y. Arima

**Affiliations:** Department of Geography and the Environment–University of Texas at Austin, 305 E 23rd St, Austin, Texas, 78712, United States of America; Universidade Federal de Goiás, BRAZIL

## Abstract

Tropical forests are now at the center stage of climate mitigation policies worldwide given their roles as sources of carbon emissions resulting from deforestation and forest degradation. Although the international community has created mechanisms such as REDD^+^ to reduce those emissions, developing tropical countries continue to invest in infrastructure development in an effort to spur economic growth. Construction of roads in particular is known to be an important driver of deforestation. This article simulates the impact of road construction on deforestation in Western Amazonia, Peru, and quantifies the amount of carbon emissions associated with projected deforestation. To accomplish this objective, the article adopts a Bayesian probit land change model in which spatial dependencies are defined between regions or groups of pixels instead of between individual pixels, thereby reducing computational requirements. It also compares and contrasts the patterns of deforestation predicted by both spatial and non-spatial probit models. The spatial model replicates complex patterns of deforestation whereas the non-spatial model fails to do so. In terms of policy, both models suggest that road construction will increase deforestation by a modest amount, between 200–300 km^2^. This translates into aboveground carbon emissions of 1.36 and 1.85 x 10^6^ tons. However, recent introduction of palm oil in the region serves as a cautionary example that the models may be underestimating the impact of roads.

## 1. Introduction

Loss of tropical forests has emerged as an important environmental issue given their importance to Earth’s nutrient and water cycles, and as repositories of biodiversity [1, 2]. Land change, primarily tropical deforestation, also account for 14–20% of global greenhouse gas emissions [3], a non-trivial amount. This has let the UN Framework Convention on Climate Change to created REDD^+^ mechanisms in an attempt to reduce emissions from deforestation and forest degradation. REDD^+^ projects typically focus on small scale forest conservation initiatives [4] but equally important is to investigate the role of regional proximate drivers of land change as an indirect source of greenhouse gases through induced deforestation. One of the most important driver of deforestation in the tropics is infrastructure development, particularly roads meant to provide access to forested areas and markets that were once inaccessible [5, 6].

The Peruvian Amazon is of particular significance in this regard because it is one of the most biodiverse areas in the world and is now the focus of several transnational transportation projects [7, 8]. Moreover, Peru is at the forefront of REDD+ initiatives and has received commitments of over US$350 million to help protect its 69 million ha of tropical and Andean forests [9]. This article addresses this issue for the Amazonian Department of Loreto by implementing a spatially explicit econometric land change model to simulate the impact of new road construction on deforestation and associated carbon emissions. The model makes use of a Bayesian spatial probit procedure developed by Smith and LeSage [10] whereby spatial dependencies are assigned to occur between groups of observations or regions instead of between individual observations, which provides a substantial computational advantage.

This article has two primary objectives, one applied and one methodological. The first is to estimate the impact of road building on the probability of deforestation and to quantify the resulting carbon emissions in Loreto, Peru, a region currently with low levels of deforestation but where infrastructure development is planned. The second objective is to demonstrate that the Smith and LeSage spatial probit model applied to a raster data environment yields better spatial allocation of estimated deforestation than standard spatially explicit probit models and therefore should be added to the portfolio of land change models. To achieve these objectives, the article begins by describing the study area and the regional context that motivates the analysis. It then shows results of a non-spatial probit model that motivates the use of a spatial probit that follows. A simulation of the impact of new roads on deforestation and carbon emissions is presented next, and the spatial and non-spatial models are compared. The paper concludes with a methodological discussion, and with an assessment of the policy implications for the Western Amazon.

## 2. Regional Context and Infrastructure Development Plans in Loreto

Located in the Western Amazon between the Equator and 9° south, the Department of Loreto is the largest in Peru, covering 368,852 km^2^. Loreto is endowed with an extraordinary aquatic and terrestrial biological diversity and is home to more than one million people, of which 12% are indigenous, belonging to 27 different groups [7, 11, 12]. National protected areas cover 18% of Loreto (67,000 km^2^), while legally titled indigenous communities account for another 4 million ha. Loreto is also rich in hydrocarbon deposits with an oil production around 8 million barrels per year. Despite this richness of resources, Loreto’s economy accounts for only 2% of Peru’s GDP and almost half of the adult population is either un- or under-employed [12].

Decision makers in the capital have long considered Loreto to be an “island state” within Peru that needs to be integrated economically to the rest of the country. Since colonial and early republican periods, natural resources from Loreto such as timber and rubber have found their way to distant markets via fluvial transportation down the Amazon River through Brazil, and to the Atlantic Ocean, 4000 km east. Although much longer, this water route was and continues to be easier and cheaper than shipping across the formidable Andes Mountains that separate the Peruvian Amazon from its coastal lands, the capital Lima, and port facilities on the Pacific Ocean. For reasons such as these, the Peruvian government has shown interest in road building, which stands to reasons since transportation infrastructure is viewed by developed and developing countries alike as fundamental to providing economic benefits and to securing sovereignty over isolated hinterlands [13, 14]. The sovereignty issues are especially relevant to Loreto, a department with a history of demands for more regional autonomy, which has been perceived by the federal government in Lima as a step toward secession. One historical episode illustrates how challenging the tenuous land connection between Lima and Iquitos was and continues to be. In 1896 the Peruvian federal government sent two military expeditions to Iquitos, Loreto’s capital, to subdue a separatist movement. News about the movement arrived in Lima only indirectly after its consulate in Rio de Janeiro, Brazil, reported the events [15]. One expedition was sent by land across the Andes and another by water along the Pacific and Atlantic coasts and Amazon River. The land expedition reached Iquitos first but the water expedition was already on the Amazon when it received orders to return to Lima. In addition to internal concerns, territorial disputes with neighboring countries along Loreto’s 3,891 km frontiers with Brazil, Colombia, and Ecuador have raised concerns about the territorial integrity of the country [15, 16]. Thus, road construction in Loreto fulfills both economic development and national geopolitical objectives [17].

**Fig 1:**
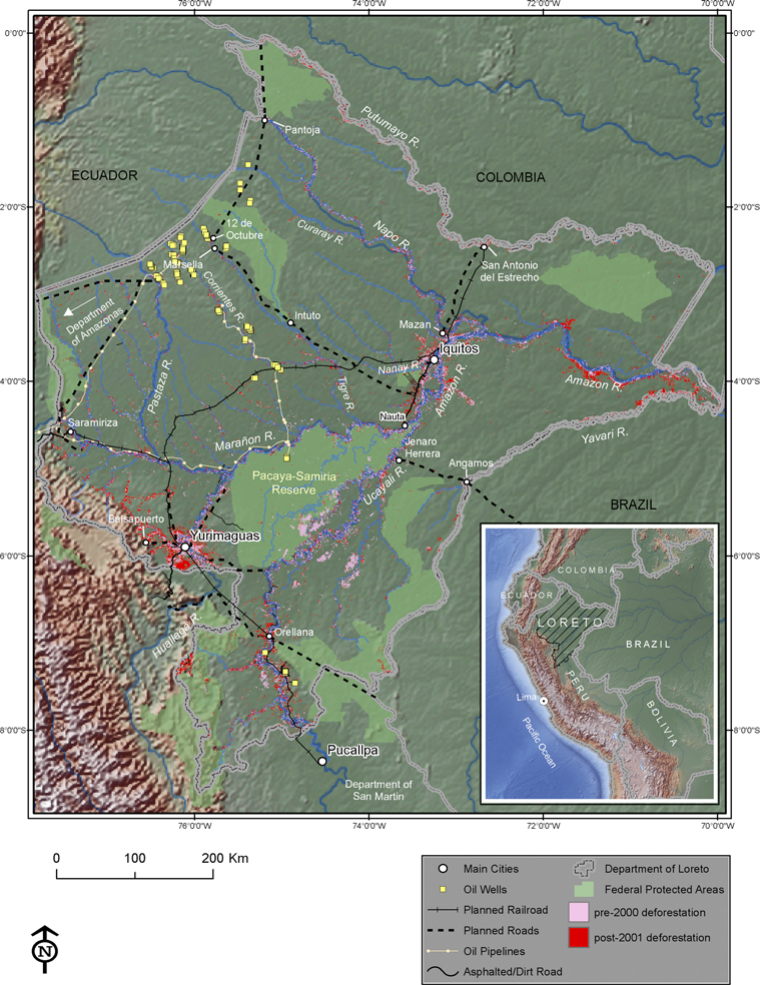
Department of Loreto in the Peruvian Amazon. Planned infrastructure will crisscross the department and will provide connection to the national road system.

The short extent of the current road network and its sparse spatial distribution illustrates the “isolated state” of much of Loreto, particularly around the capital Iquitos (Fig 1). According to GIS data [18], only two road segments comprising ~200 km are asphalted. One segment connects the city of Yurimaguas in the western part of Loreto to the national road network at Tarapoto; this was asphalted in 2009 as part of the Integration of Regional Infrastructure in South America (IIRSA) Norte program [19]. The other asphalted road links the capital Iquitos to an important local port in Nauta at the confluence of the Marañon and Ucayalli Rivers. Year round trafficable dirt roads are roughly 130 km in length while intermittent dirt roads and trails add another 823 km to the network. Roads built to service oil pipelines amount to 400 km, but are not used for general transportation because they remain detached from the network.

Roads have long been considered an important driver of deforestation in many tropical countries [5, 20]. In Peru, 75% of total deforestation and forest degradation is within a 20-km buffer along roads [21]. This road-deforestation nexus has also been observed recently with the road construction and paving of IIRSA’s Inter-Oceanic Highway in the department of Madre de Dios, which have induced new pulses of deforestation [8]. Nevertheless, perhaps because of its sparse road network, less than 5% of Loreto is deforested (~17,500 km^2^), mostly for low intensity swidden agriculture based on cassava, plantains, rice, and maize, although palm oil plantations are beginning to encroach on the region [22]. Deforestation is currently concentrated along the Iquitos-Nauta road, near the city of Yurimaguas, and in communities found along Loreto’s extensive hydrologic network [12] (Fig 1).

Concerns about extensive deforestation in Loreto emerged in 2005 when the Peruvian government, together with the regional government, released a transportation development plan for the department [23]. The plan calls for a series of multi-modal investments including the construction of a railway connecting Iquitos with Yurimaguas, upgrade of port facilities and airports, dredging of rivers, and waterways signalization. The plan also includes the construction of 1,780 km of new roads that will provide, together with waterways and railways, connections between important national and regional hubs such as Iquitos, Yurimaguas, Pucallpa, Saramiriza, and strategic communities bordering Colombia and Brazil, and oilfields close to Ecuador (Fig 1).

Despite these ambitious development goals, only a few qualitative studies have been conducted regarding potential environmental impacts of such projects. Recent studies have projected that deforestation will reach 1.8 million hectares in 2021 or 4.8% of Loreto, mostly “along new roads” [12]. These projections are based on linear extrapolation of past trends and although these numbers are feasible, modeling efforts to date have been limited in their capacity to predict the spatial distribution and pattern of future deforestation. This article fills this gap by assessing the impact of new roads in the region on deforestation through a raster-based spatial econometric model, a topic addressed in the following sections.

## 3. Conceptual Model of Deforestation and Data Set

The conceptual model aims to explain deforestation by considering the role of market access to agricultural land use decisions. Key to this model is the concept of potential economic rent developed by von Thünen, whereby rent declines as a function of transportation costs. In this model, agents are more likely to deforest when rents from agricultural land uses (R^a^) are greater than rents obtained from standing forests (R^*f*^), both being non-negative [24, 25].

Rents for a given pixel *i* depend of the prices obtained for agricultural or forest products net of transportation costs and therefore can be stated as functions of Euclidean or weighted distances from transportation corridors such as rivers, roads, and central markets [24, 26]. The likelihood of a given pixel *i* being deforested is then modeled as a linear function of (weighted or unweighted) distances (*TC*) and other control variables that affect rent such as protection status, biophysical characteristics, and socioeconomic variables (**x**). Define y*_i_ = R^a^_i_—R^f^_i_ = *αTC*_*i*_ + **x**_*i*_**β** + *e*_*i*_. Assuming agents are profit-maximizers, the probability of observing deforestation (*y = 1*) can be written as:

$$P(y=1|x,TC)=P(y^{*}>0|x,TC)=P(e>-\alpha TC-x\beta |x,TC)=\Phi (\alpha TC+x\beta )$$

$$y_{i}=\left\{ \begin{array}{c} 0,\;if\;y^{*}<0\\ 1,\;if\;y^{*}>0 \end{array}\right.$$

where *α* is the vector of coefficents associated with *TC* and **β** the vector of coefficients associated with the vector of controls **x**. If we assume that the unobservable term *e* is normally distributed, then the probability of deforestation is given as *P*(*y* = 1|**x**, TC) = *P*(y* > 0|**x**, TC) = *P*(*e* > −*α*TC − **xβ**|**x**, TC) = Φ(*αTC* + **xβ**), where Φ(^.^) is the cumulative normal density function, which leads to a non-spatial probit model.

### 3.1. Road endogeneity issues

The proper identification of the effect of roads on deforestation must address potential endogeneity in the placement of roads, a routing decision that can arise from two processes. First, roads can be purposefully routed through areas with higher suitability for agricultural development (e.g. better soils, terrain, and climate) and if such biophysical factors are left uncontrolled, the effect of roads on deforestation will likely be overestimated [24, 27]. The second case arises from an inverse causality process whereby deforestation actually precedes the construction of roads, which are therefore built as a response to agricultural development [28]. For the case of Loreto and this analysis in particular, where only three year-round trafficable roads exist, endogeneity does not seem to be likely. As explained above, the construction of the Yurimaguas -Tarapoto road was motivated mostly by national and international interests in connecting two large cities to the national network system in order to facilitate the transportation of goods [19]. The road connecting the upper Marañon River in western Loreto to the national grid was motivated by oil exploration in that region and is therefore unrelated to agricultural development objectives [29]. Finally, the Iquitos-Nauta road construction was motivated mostly by the savings in transportation time between Loreto’s capital and this important logistic point at the confluence of the Marañon and Ucayalli Rivers, which are the main transportation arteries in Loreto [30]. In addition, the regression analysis (see below) attempts to control for biophysical characteristics that may be correlated with deforestation and road construction such as soil type, precipitation, ecosystem type, and presence of wetlands. The analysis also masks out all deforestation that occurred prior to 2000, the year when all three trafficable roads currently in existence in Loreto became fully connected during the dry season with the completion of bridges, although the opening of the road paths began in the 1970s in fits and starts and paving was completed only in the mid-2000s [30, 31]. While endogeneity cannot be ruled out completely, the empirical context and the analytical setup work to mitigate these concerns.

### 3.2 Long-term effect of roads on deforestation

In addition to endogeneity issues, another modeling challenge arises by virtue of the long term effect of roads on deforestation. Typically, policy makers, Earth systems scientists, and ecologists are interested in the accumulated total effect, not on yearly rates. However, total deforestation due to road construction is not observed instantaneously in a single period after construction; rather its effects are observed over a duration lasting years. This is because we rarely observe landholders clearing all their plots in a single time [32], although theory predicts that in steady-state equilibrium, the sum of annual rates will reach an optimal total amount of deforestation [33]. As stated previously, I use observed deforestation accumulated from 2001–2014 in the estimation. The upper bound year (2014) is the most current deforestation estimate obtainable from satellite images. Thus, the analysis presented here can be interpreted as the accumulated effect of roads on deforestation in a 14 year period after a road acquired a status of “fully passable.”

### 3.3 Model variables and data sources

The data used in the statistical analyses were made available to the author by the Wildlife Conservation Society (WCS) office in Peru; they were also derived from online sources. All digital files were either in or converted to raster format, projected to UTM coordinate system, and resampled to 900 m cell resolution using nearest neighbor algorithms. This meso-scale cell resolution was chosen as a good compromise among the various scales of the data available. For instance, the deforestation data (see below) is at 30 m resolution, whereas the ecosystem maps represent units at scales of “thousands of hectares” [34]. After masking out previously deforested cells (pre-2000) and open water cells, these 900m cells constitute the units of observation (n = 435,138). The dependent variable ‘deforestation’ is from the Peruvian Ministry of the Environment and Ministry of Agriculture [35]. Originally at 30 m resolution, this Landsat-based dataset detected deforestation prior to 2000, the baseline year, and from 2001to 2014 on an annual basis. According to this dataset, accumulated deforestation until 2000 was 9,142 km^2^ and from 2001–2014 the area deforested amounted to 8,330 km^2^, resulting in a total deforested area of 17,472 km^2^ or 4.6% of Loreto.

In most spatially explicit models, rent is proxied by Euclidean distances to roads, to navigable rivers, and to main cities [36, 37]. These variables were calculated within ArcGIS^®^ using built-in functions; the average distance of the pixels to a road segment was 155.8 km (standard deviation, or s.d. 90.8 km) with a maximum distance of 477 km (Table 1). The average distance to a navigable river was much smaller, only 7.77 km (s.d. 6.1 km), an indication of the extensive hydrologic network in Loreto. The average distance of the pixels to the nearest city in the region, either Iquitos, Yurimaguas, or Pucallpa was 191.5 km (s.d. 86.6 km) with a maximum distance of 480 km.

**Table 1:** Descriptive statistics of the variables used in the regression (n = 435,138).

Variable (abbreviation between parentheses)	Unit	Mean	Std. Dev.	Min	Max
Deforestation	binary	0.026	0.158	0	1
Distance to nearest road (Droad)	kilometers	155.782	90.810	0	477.00
Distance to nearest river (Driver)	kilometers	7.767	6.074	0	43.879
Distance to nearest city (Dcity)	kilometers	191.473	86.563	0	480.038
Accumulated cost (Wdist)	Soles/sack	43.329	34.999	0	184.764
Distance to pre-2000 deforestation (Ddft)	kilometers	12.628	10.136	0.9	57.240
National Protected Areas (NPA)	binary	0.181	0.385	0	1
Elevation (Dem)	meters	185.72	155.16	56	2182
Slope (Slp)	percent	0.936	2.573	0	58.872
Precipitation (Precip)	meters/yr	2.901	0.465	1.547	3.584
Gleysols (Gley)	binary	0.372	.483	0	1
Cambisols (Camb)	binary	0.163	0.369	0	1
Alisols (Alis)	binary	0.047	0.211	0	1
Acrisols (Acri)	binary	0.403	0.490	0	1
Distance to pipeline (Dpipes)	kilometers	175.784	133.692	0	568.340
Distance to oil wells (Dwells)	kilometers	161.800	125.913	0	565.172
Black water environment (Blkwt)	binary	0.244	0.429	0	1
White water environment (Whtwt)	binary	0.044	0. 206	0	1
Non-flooded forests (Ralta)	binary	0. 558	0. 496	0	1
Wetlands (Wet)	binary	0.122	0.328	0	1

In addition to unweighted Euclidean distance variables, I specified a model with a variable representing ‘accumulated transportation costs’ (Wdist_*i*_) that captures the weighted distance between each cell *i* to the nearest market place. Specifically, Wdist_*i*_ = min(Wdist_*i1*_, Wdist_*i2*_, Wdist_i3_), where the indexes 1, 2, 3 denote the three relevant market centers in the study area, Iquitos and Yurimaguas in Loreto, and Pucallpa in the Department of San Martin (Fig 1). The transportation cost between each cell and a market center was calculated within ArcGIS^®^ using cost distance functions based on the Dijkstra algorithm [38]. I first assign a “friction value” for each cell, which is the cost of traversing the cell (Table 2). The friction value varies according to the mode of transportation (e.g. river, road) and quality and type of infrastructure (e.g. paved road vs. dirt road or large river vs. narrower, smaller river). For example, transport of one sack of grain/fruits (50–60 kg) by boat along a large river such as the Ucayali costs $0.0103 km^-1^ and $0.30 km^-1^ on a dirt road (values in Peruvian Soles). In essence, lower friction values “shorten” the size of the cell relative to cells with higher friction values. Thus, two cells of same resolution may have different weighted “lengths” depending on their friction value. These transportation costs were based on 42 surveys conducted by a WCS staff member during the low water season of 2014 along the Ucayali and Maranõn Rivers; they were also taken from the literature (see sources on Table 2). Hence, the accumulated transportation cost variable is a weighted distance measurement that incorporates information about distance to markets as well as transportation costs. The calculated accumulated cost values range from $0 to 184/sack, with a mean of $43.33, a median of $32, and a s. d. of $35 (Table 1).

**Table 2:** Transportation costs according to different modes of transportation and infrastructure quality.

Type	Friction ($/meter/sc)	Source
Class I Rivers	0.0000103	From WCS survey
Class II Rivers	0.0000320	From WCS survey
Class III Rivers	0.0000534	From WCS survey
Class IV Rivers	0.0000750	From WCS survey
Asphalted Roads	0.0001000	Roughly 30% more expensive than class IV rivers (Arima et al. 2007)
Dirt Road (trochas)	0.0003000	3x more expensive than on asphalted road (Arima et al. 2007)
Natural bed—forest	0.0020000	20x more expensive than on asphalted road (Arima et al. 2007)

A set of dummy variables was also included to control for different biophysical characteristics. I used NatureServe’s ecosystems macrogroup classification to distinguish white (4.4% of the area) and black water flooded forests (24.4%), as they may have different agricultural aptitudes due to differences in river sediment deposition [34, 39]. Black water ecosystems include the classes ‘black water floodplain forests of the Western Amazon, black water flooded forests of the Western Amazon, black water flooded forests of the Southwestern Amazon.’ White water ecosystems are ‘white water floodplain forests of the Western Amazon, white water flooded forests of the Western Amazon, white water flooded forests of the SouthWestern Amazon, and white water flooded forests of the Upper Amazon. Non-flooded forests include ‘forests of the plains of the Western Amazon, forests of the peneplains of the Western Amazon, and forests of the peneplains of the SouthWestern Amazon. Subandean forests constitute the omitted variable.

Lowland, non-flooded forests occupy 55.8% of the area and sub-Andean forests (omitted categorical variable) account for the remaining 15.4% (Table 2). In addition to those ecosystem variables, a binary variable that identifies wetlands [40, 41] was also included because these environments subject to annual flooding are less likely to have roads built on them. Approximately 12% of Loreto’s area is wetland, located mostly along the rivers and between the Marañon and Ucayalli rivers, a geologic region known as the Ucamara depression. Also included was elevation from the Shuttle Radar Topography Mission and derived slope variables. Loreto’s mean elevation excluding masked cells is only 185.7 m but it ranges from 54 to 2,182 m in its western Andean portion. Likewise, the average slope is only 0.9%, but ranges from 0 to 58.9% or roughly 30 degrees. Completing the biophysical controls, I added soil type and precipitation since both have been shown to be associated with deforestation and agricultural productivity in Amazonia [32, 42]. Precipitation data are from the Tropical Rainfall Measuring Mission (TRMM) product 3B43 version 7. Through the use of raster algebra, monthly rainfall values per hour were converted to a 16-year (1999–2014) average rainfall raster. Average rainfall in Loreto is 2.901 meters/yr with a range between 1.547–3.584 m and s.d. of 0.465 m. Soil data are from the harmonized world soil database version 1.2 [43]. The soil types include gleysols (37%), cambisols (16%), alisols (4.7%), and acrisols (40%). The omitted category contains leptosols and a small portion of fluvisols.

Two other variables, Euclidean distance to oil wells and pipelines, control for the potential effect of the oil industry on deforestation. The average distance of the cells to a pipeline and well is 175 and 162 km respectively, with a maximum value at around 568 km. Distance to pre-2000 deforested areas was also included in some model specifications because new deforestation tends to sprawl from old deforestation. Moreover, this variable also control for other unobservables that may be correlated with roads [42]. Finally, a dummy variable was included to identify cells inside national protected areas, which account for approximately 18% of Loreto.

## 4. Spatially Explicit Models

### 4.1 Non-spatial probit model

Before presenting the spatial probit model application developed by Smith and LeSage [10] (henceforth SLS), a non-spatial probit (henceforth NSP) model is first considered in order to provide a context and motivation for the implementation of the spatial model. Table 3, shows NSP regression results using the explanatory variables described in the previous section, with Huber-White robust standard errors in parentheses. Model 1 (NSP1) uses Euclidean distance measures in level and squared values as explanatory variables in addition to the other control variables. Model 2 (NSP2) uses the weighted distance variable (Wdist) instead of the Euclidean distance variables. Interpretation of results relies not only on statements about statistical significance but also on the practical significance of each variable. I thus measure the average partial effect (APE) for a continuous variable *x*_*k*_ in levels and quadratic form as $N^{-1}\sum_{i=1}^{n}\left({\hat{\beta }}_{k}+2{\hat{\beta }}_{k^{2}}x_{ik})\phi (x_{i}\hat{\beta }\right)$ where *ϕ* is the standard normal density function and ${\hat{\beta }}_{k}$ is the estimated coefficient of the variable in level form and ${\hat{\beta }}_{k^{2}}$ the estimated coefficient of the variable in quadratic form. APEs for a variable without its quadratic form are obtained by setting ${\hat{\beta }}_{k^{2}}=0$. For a binary variable *k*, APE is $N^{-1}\sum_{i=1}^{n}\left[\Phi (x_{k-,i}{\hat{\beta }}_{k-}+{\hat{\beta }}_{k}\right)-\Phi (x_{k-,i}{\hat{\beta }}_{k-})]$, where the index *k-* indicates that the variable *k* is not part of the vector and Φ is the cumulative normal. Standard errors for the partial effects were also calculated but not reported since the effects were all statistically significant at p = 0.01, with the exception of elevation and slope partial effects (significant at p = 0.05).

**Table 3:** Regression results for non-spatial probit (NSP) and SLS spatial probit.

Variable	Coefficient(standard error)				
	NSP1	NSP2	SLS1	SLS2	SLS3
Wdist		-0.0052***			-0.0102***
		(0.0002)			(0.0006)
NPA	-0.351***	-0.363***	-0.5335***	-0.5355***	-0.5562***
	(0.019)	(0.0193)	(0.0542)	(0.0544)	(0.0236)
DEM	-0.0002*	-0.0005***	-0.0027***	-0.0026***	-0.0040***
	(8.9e-05)	(0.0001)	(0.0001)	(0.0001)	(0.0001)
Slope	0.0067**	0.0149***	0.0502***	0.0503***	0.0795***
	(0.0032)	(0.0031)	(0.0056)	(0.0056)	(0.0071)
Dpipes	0.0031***	0.0036***	0.0037***	0.0037***	0.0007***
	(0.0002)	(0.0002)	(0.0003)	(0.0004)	(0.0001)
Dpipes^2^	-9.69e-06***	-9.41e-06***	-1.2e-05***	-1.1e-05***	
	(7.38e-07)	7.30e-07	(1.0e-06)	(1.0e-06)	
Dwells	-0.0020***	-0.0001	-0.0016***	-0.0016***	0.0018***
	(0.0002)	(0.0002)	(0.0004)	(0.0004)	(0.0002)
Dwells^2^	1.10e-5***	6.19e-06***	1.1e-05***	1.1e-05***	
	(7.79e-07)	7.31e-07	(1.0e-06)	(1.0e-06)	
Ddft	-0.1351***	-0.159***	-0.2495***	-0.2495***	-0.1334***
	(0.0023)	(0.0024)	(0.0089)	(0.0087)	(0.0167)
Ddft^2^	0.0025***	0.0030***	0.0048***	0.0048***	
	(4.38e-05)	(4.38e-05)	(0.0002)	(0.0002)	
Droad	-0.0023***		-0.0045***	-0.0045***	
	(0.0002)		(0.0007)	(0.0007)	
Droad^2^	6.99e-06***		1.4e-05***	1.4e-05***	
	(8.67e-07)		(2.0e-06)	(2.0e-06)	
Driver	-0.0894***		-0.2213***	-0.2226***	
	(0.0028)		(0.0087)	(0.0086)	
Driver^2^	0.0024***		0.0060***	0.0060***	
	(8.44e-05)		(0.0002)	(0.0002)	
Dcity	-0.0038***		-0.0057***	-0.0057***	
	(0.0003)		(0.0005)	(0.0005)	
Dcity^2^	5.47e-06***		9.0e-05***	9.0e-05***	
	9.31e-07		(1.0e-05)	(1.0e-05)	
Precip	-0.330***	-0.330***	-0.4692***	-0.4664***	-0.5217***
	(0.0180)	(0.015)	(0.0303)	(0.0304)	(0.0432)
Blkwt	-0.488***	-0.516***	-0.7648***	-0.7643***	-0.7960***
	(0.0180)	(0.017)	(0.0259)	(0.0259)	(0.0392)
Whtwt	-0.140***	-0.118***	-0.3376***	-0.3373***	-0.1134***
	(0.0195)	(0.019)	(0.0358)	(0.0358)	(0.0465)
Ralta	-0.135***	-0.271***	-0.1977***	-0.1995***	-0.3972***
	(0.020)	(0.0184)	(0.0221)	(0.0221)	(0.0340)
Wet	-0.190***	-0.196***	-0.2048***	-0.2033***	-0.1931***
	(0.0153)	(0.0153)	(0.0230)	(0.0231)	(0.0206)
Gley	0.722***	0.407***	-0.2555***	-0.2492***	-0.6985***
	(0.085)	(0.0812)	(0.1075)	(0.1084)	(0.1168)
Camb	0.752***	0.529***	-0.0656	-0.0633	-0.3747***
	(0.080)	(0.077)	(0.0932)	(0.0937)	(0.0976)
Alis	0.669***	0.312***	-0.3127**	-0.3090*	-0.5831*
	(0.094)	(0.0912)	(0.2008)	(0.2023)	(0.1250)
Acri	0.661***	0.419***	-0.3417***	-0.3359***	-0.6428***
	(0.0864)	(0.0825)	(0.1174)	(0.1187)	(0.1210)
constant	0.0075	-0.4605***	2.135***	2.1168***	1.0653***
	(0.100)	(0.0962)	(0.124)	(0.1254)	(0.1638)
*ρ*			0.0475***	0.1036***	0.1500***
			(0.0190)	(0.0313)	(0.0223)

Note

*^,^ **^,^ *** significant at p< 0.1, p<0.05, p<0.01 respectively; n = 435,138.

The variables of interest, distance to roads and ‘transportation cost’ (Wdist), have the expected negative sign for the level term and are both statistically significant in both NSP1 and NSP2. The coefficient for the quadratic term for distance to roads is positive in the NSP1 model, indicating that the combined effect becomes smaller as distance increases. The estimated average partial effect (APE) for Wdist is relatively small; a 1 Peruvian Sol increase in freight cost will decrease the probability of deforestation by 0.0003. Likewise, a one kilometer increase in distance to a road decreases the probability by an average of 3.0e^-5^. The APE of distance to rivers is two orders of magnitude larger, -0.0032, indicating the importance of rivers as the main transportation mode in the region. On the other hand, distance to the nearest city had a small APE of -1.0e^-4^ (in model NSP1). National protected areas (NPA) are also highly significant and reduce deforestation by 0.016 (in NSP1) and 0.017 (in NSP2), a surprisingly large effect given that deforestation is quite low even outside protected areas.

The impact of the physical variables on deforestation varied in magnitude. Although all variables are statistically significant, elevation (DEM) and slope APEs are quite small (APE 10^−6^ and 10^−4^ order of magnitude respectively). The ecosystem type variables had much larger APEs on both NSP1 and NSP2: deforestation is 0.023 smaller in black water (Blkwater) and 0.06 in lowland non-flooded (Resalta) environments on average, when compared to the omitted category sub-Andean forests. White water environments also showed small negative APEs around -0.006 on both models, effects similar to wetlands (-0.009). The APEs of soil types on deforestation are all positive with respect to the omitted variable leptsols, with a 10^−2^ order of magnitude. Finally, precipitation also had a small negative effect on deforestation with APEs of -0.15 in both models (the unit is in meters of rain per year).

Oil exploration does not seem to impact deforestation. Distance to oil wells (Dwells) and distance to pipelines (Dpipes) had opposite effects and negligible APEs values, similar in magnitude to elevation (10^−5^). Overall, the estimated probabilities range from 0.0 to 0.486 (NSP1) with an average of 0.0256, a value similar to the overall proportion of deforested cells in the dataset. For the NSP2 model, predicted probabilities range from 0.0 to 0.374 with the same mean probability value. Different model specifications without squared variables yielded similar estimates, and results are available upon request to the author.

**Fig 2:**
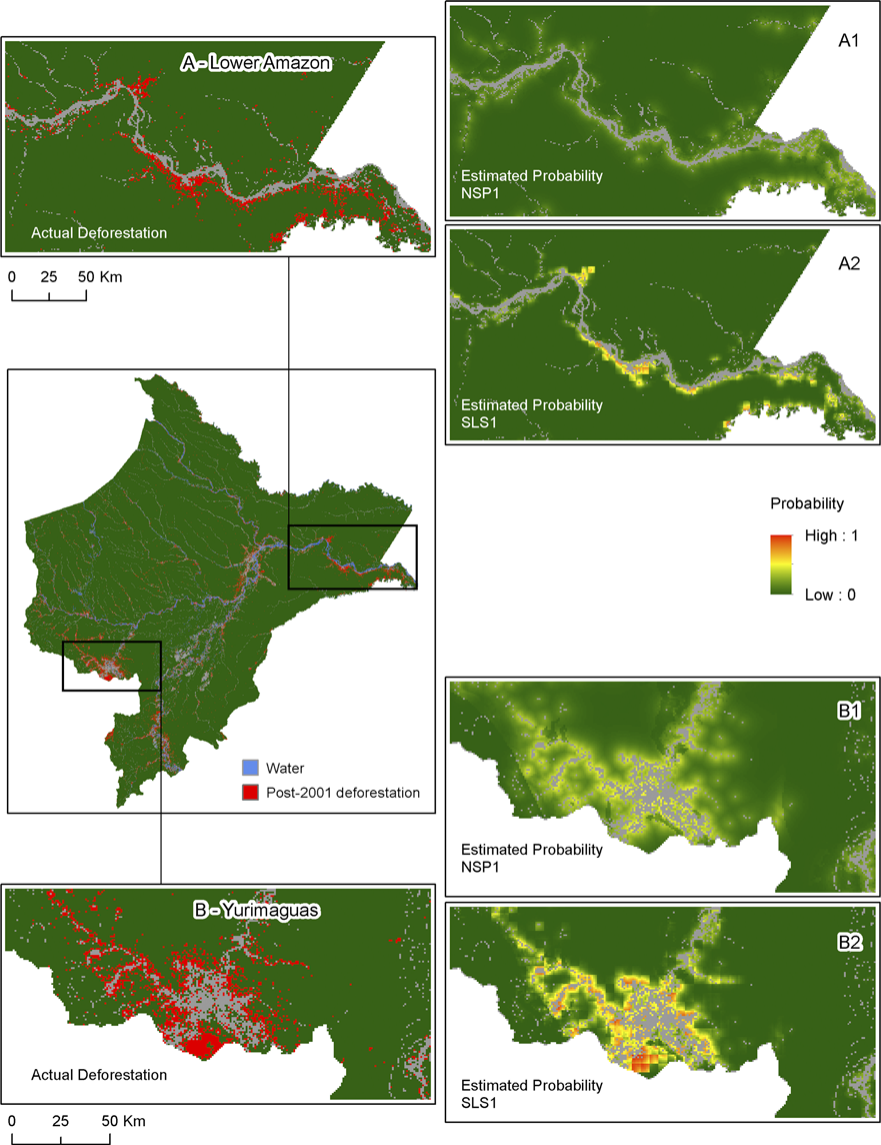
Actual deforestation and estimated probabilities. (A) Actual deforestation along the lower Amazon River from 2001–2014. (B) Actual deforestation near Yurimaguas from 2001–2014. (A1 & B1)–Estimated probabilities from NSP1 model. (A2 & B2) Estimated probabilities from SLS1 model.

**Fig 3:**
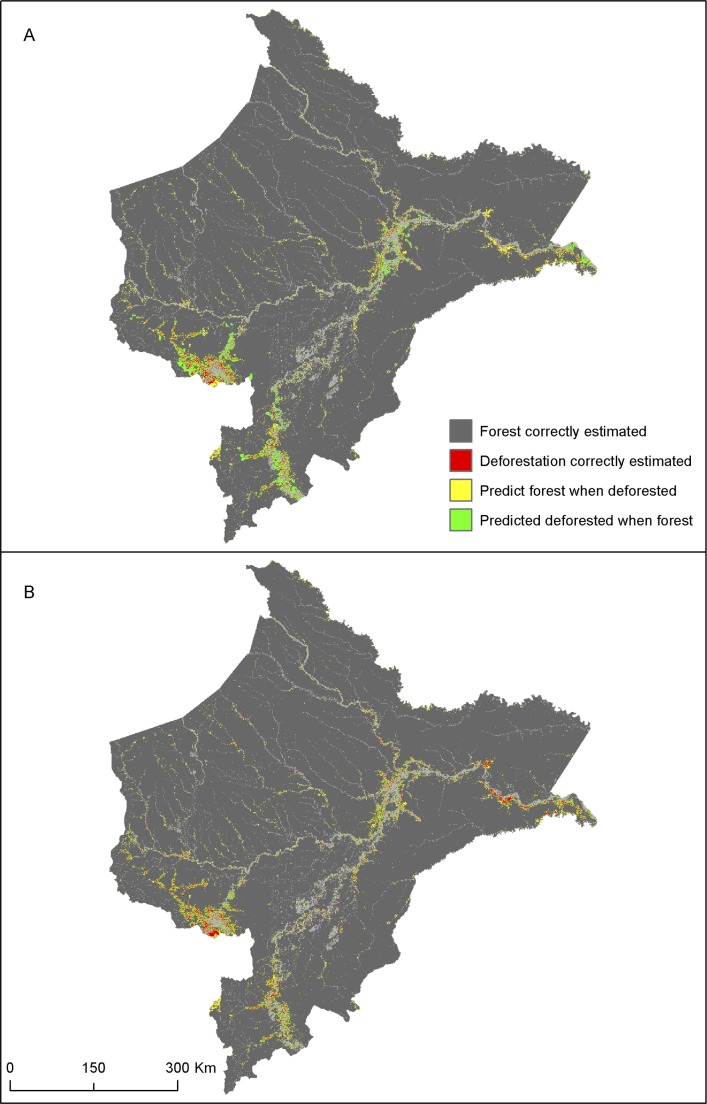
Cross tabulation of actual vs. predicted deforestation. NSP1 Model. (B) SLS1 Model spatial allocation of deforestation is superior to the NSP1 Model.

In addition to issues of spatial autocorrelation that can lead to parameter bias and/or inefficient standard error estimation [44], NSP models usually fail to replicate complex spatial patterns of land change, which is an important consideration since ecology and Earth system sciences are interested not only in the amount of deforestation but also where deforestation is likely to occur. Consider the maps shown in Fig 2; here, the left central panel shows actual deforestation in Loreto in red and panels A and B show details of two regions, the Lower Amazon and Yurimaguas. Estimated probabilities are shown on the right panels and are color coded with low probabilities of deforestation in green and high probabilities in red. Since the maximum estimated probability of the NSP1 model is 0.48, all cells in A1 and B1 are in green hue. In order to examine location agreement/disagreement patterns, cells predicted to be deforested were cross tabulated with actual deforested cells (Fig 3). The cells predicted to be deforested were selected by searching for a threshold probability p* such that the sum of the areas of cells *j* with estimated probability ${\hat{p}}_{j}\geq p^{*}$ equals the observed amount of deforestation [45]. This probit goodness-of-fit measure is preferred when one outcome is unbalanced in the sample, which is the case of Loreto where only 2.5% of the cells are deforested [45]. The calculated threshold probability p* was 0.181. That is, the number of the cells with estimated probability greater than 0.181 equals the observed 11,218 cells that are actually deforested. Thus, by fixing the estimated area to match the actual value, we can investigate how well the model allocates deforestation spatially. Predicted deforestation (cells in red and green) is clustered along the Ucayalli, Amazon, lower Yavari, parts of Marañon, and Napo rivers, where probabilities are higher due to cheaper fluvial transportation costs and proximity to the three regional market centers, and also because this distance-based variable is multiplied by one single global parameter $\hat{\alpha }$. The NSP model fails to capture the fragmented nature of deforestation in Loreto as evidenced by the cross tabulation of actual and predicted deforestation in Fig 3, panel A. Approximately 30% (3,316 cells) of the actual deforestation was correctly allocated by the model (cells in red). The remaining 7,902 cells (70%) were areas that are actually deforested but were predicted to be forests (in yellow). These under-predictions are concentrated around Iquitos and Yurimaguas, lower Amazon River as well as in isolated pockets of deforestation along smaller rivers. Since the deforestation quantity is fixed to match actual deforestation, an equivalent area was (over-) predicted as deforested when in fact it is forested (in green).

To summarize, the non-spatial probit model generates probability surfaces that are usually very smooth, with little local variation because it does not incorporate any spatial neighborhood effects. Thus, fragmented patterns of deforestation are not easily reproduced or captured by such models. This is a significant shortcoming when the objective of the model is not only to statistically determining the drivers of land change but also to predict patterns of landscape change or fragmentation that may be important to certain ecological and biophysical processes (e.g. local extinction of species, viability of fragments, fire regime, carbon emissions).

### 4.2 Spatial probit regression model

Smith and LeSage [10] developed a probit model with spatial dependencies where individual observations are assigned to regions. In their model, spatial dependencies are ascribed to occur between regions, not between individual observations. In their model implementation, SLS used county-level observations grouped by states (i.e. regions) and spatial dependencies were assigned between states instead of between individual counties. Following SLS, we can write our empirical model of deforestation for pixel *i* in region *j* as:

$$y_{ij}^{*}=\alpha {TC}_{ij}+x_{ij}\beta +\theta_{j}+e_{ij}$$

$$\theta_{j}=\rho \sum_{k=1}^{m}w_{jk}\theta_{k}+\mu_{j}$$

where *θ*_*j*_: *j* = 1, …, *m* is the regional effect and is defined as a spatial autoregressive process, where *w*_*jk*_ are the weights reflecting the degree of spatial ‘closeness’ between regions *j* and *k*, and *ρ* is the parameter that reflects the overall degree of spatial dependency between regions. In essence, this model contains two unobserved components, a region specific effect *θ*_*j*_ and an individualistic effect *e*_*ij*_. The regional effect *θ*_*j*_ captures unobserved phenomena that are constant for all pixels *i* ∈ *j* [44]. The idiosyncratic error *e*_*ij*_ is assumed to be normally distributed, conditional on *θ*; *μ*_*j*_ is also assumed to be normally distributed.

**Fig 4:**
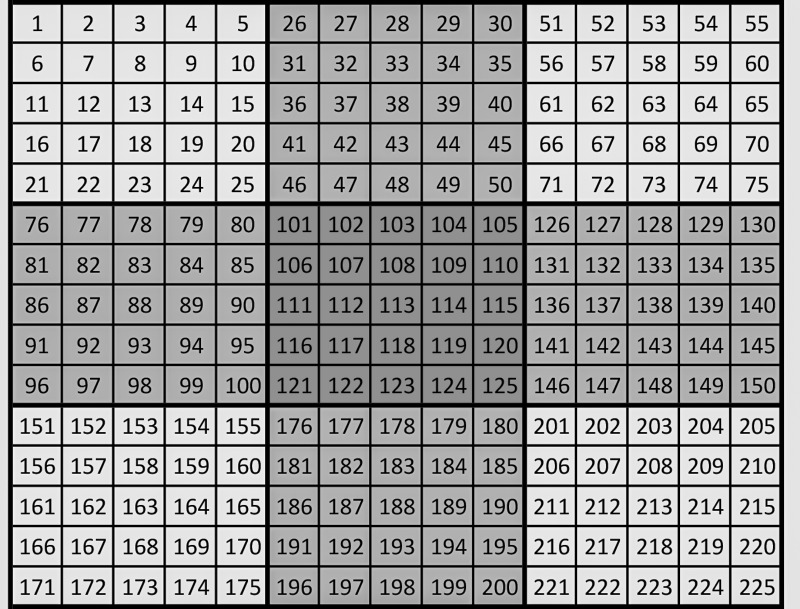
Raster implementation of regions and spatial neighborhoods. In this example, region in the center is neighbor to the four rook neighbors in light gray.

To implement this model in a raster format, two Matlab scripts were written. The first script divides the raster extent into regions that are 5x5 cells in size, labels and sorts each pixel to its region, and creates a file containing the number of pixels within each region, which in this application can be less than 25 for regions near the border of the raster extent or if a region contains cells with masked out values. This 5x5 region size was chosen because it yields a good compromise between computational feasibility (smaller regions are more computationally demanding) and individual specificity (larger regions assume same unobserved effect over a larger area). The second script creates regional neighborhood weight matrices and normalizes each row sum $\sum_{k=1}^{m}w_{jk}$ to one while keeping track of the pixel *i* region *j* sorting. Three model specifications are shown in Table 2. Models SLS1 and SLS3 use neighborhood matrices where only the four nearest neighboring regions, equally weighted, are considered. Model SP2 uses a neighborhood matrix whereby the weights for each region are calculated proportionally to the inverse of the distance to the nearest 24 regions. Fig 4 shows the example of the four nearest neighbor case for a simple raster with cells labeled 1–225 divided into 9 regions, each with 25 cells. The region in the middle containing cells 101–125 and colored in dark gray has four neighboring regions colored in light gray.

Spatial econometric models have rarely been used with raster or gridded data when the extent of the study area is large as is the case of Loreto, because of the significant computational challenges associated with the high dimensionality of the corresponding spatial weight matrix *W* when spatial dependencies are assigned between each individual observation. Fig 4 exemplifies why the SLS model is computationally less demanding than a model where spatial dependency is individually assigned. In the latter case, the neighborhood matrix for this small raster would be 225x225 in size. The SLS model would only require a 9x9 neighborhood matrix.

For the Loreto area, the scripts allocated the 435,138 raster cells into 18,903 regions and created a corresponding (sparse) weight matrix of similar size. To run the SLS probit regression, the function *semip_g* from LeSage’s spatial econometric Matlab library was used [46]. This function implements a hierarchical Bayesian approach and a Markov Chain Monte Carlo (MCMC) sampler to estimate the parameters of interest. The results presented in Table 3, columns 4–6, are based on the average of 500 valid draws after the first 500 were omitted for convergence (burn-in) of the MCMC sampler. Likewise, the reported standard errors between parentheses are the standard deviation of the last 500 draws. In the Supplementary Information document (S1 File), the parameters and distributions used in the sampler are summarized but readers are encouraged to find detailed description of the methods in the original manuscript by [10, 44]. The average partial effects for the SLS model are calculated similarly to the NSP case because it does not contain any spatial spillover effect [44]. The only difference is that now the latent variable’s fitted values include the regional spatial effect.

The estimated coefficients for the SLS1 and SLS2 are essentially the same with minor variation at the fourth decimal place, indicating that the different neighborhood matrices had little impact on the estimation, a finding consistent with recent literature [47]. The discussion below will focus on SLS1, which uses the same variables as the NSP1 model. The sign of the coefficients of the spatial probit models agree with the NSP estimates with the exception of the soil variables, which are positive in the NSP models and negative and statistically significant in the SLS model. The APE for distance to roads is -3.59e^-05^ and very similar to the NSP1. APE for distance to rivers is also in accordance with the NSP1 model, -0.0038. Similarly, distance to nearest city yielded an APE of -2.0e^-5^. SLS3 estimated an APE for Wdist smaller than the estimated non-spatial probit and indicates that a 1 Sol. increase in freight cost will decrease the probability of deforestation by -0.0002. The APE of national protected areas is -0.0083, still a relatively sizeable impact. The spatial effects parameter *ρ* indicates positive and significant spatial dependence between regions, as expected.

The advantages of the spatial over the non-spatial model for land change models are clearly visible in Fig 2. Panels A2 and B2 use the same color code as in A1 and B1 to show estimated probabilities, which now range from 0 to 0.98. Patterns and clusters of high (and low) deforestation probabilities are discernible due to the strong spatial regional neighborhood effect. Clusters of high probability near Iquitos, Yurimaguas, lower Amazon, and upper Ucayali River match actual deforestation reasonably well (Panel A). Fig 3, panel B replicates the observed quantity of deforestation by defining a cell *j* to be deforested if ${\hat{p}}_{j}\geq p^{*}=0.160$. The ‘blocky’ appearance of the map reflects the strong regional effect whereby the cells within each block show similar magnitudes in probabilities. Unlike NSP, deforestation estimates are not continuous over the landscape but show a more fragmented and jagged pattern, consistent with actual deforestation. The cross tabulation (Fig 3, panel B) indicates that 44% of the actual deforestation was correctly allocated by the SLS model, a large improvement when compared to the NSP (30%), although 56% of deforested cells are still either over or under-predicted (as deforested).

The better performance of the SLS model in allocating predicted deforestation is confirmed by Hagen’s fuzzy similarity metric [48]. The cross-tabulations shown in Fig 3 and statistics such as Cohen’s Kappa coefficient measure the degree of similarity between two rasters by evaluating if the state of a cell (e.g. deforested or forested) in the predicted raster perfectly overlaps the actual raster. This pixel-by-pixel comparison is problematic because it treats a small offset error in location, say by one cell, the same as a large displacement error [49]. Therefore, similarity metrics that bring cell neighborhoods into the calculation are preferred. Following [50], I coded Hagen’s Two-Way fuzzy similarity metric using a neighborhood weight matrix based on an exponential decay function (W = 2^-d/2^), where *d* is the distance between the cell center and each of the neighborhood cells within a defined window kernel. This neighborhood weight matrix incorporates fuzziness in location and measures the degree of similarity of the simulated center cell to its corresponding center cell and neighbors of the actual raster. The Two-Way fuzzy metric ranges from 0 to 1, where 1 depicts a perfect center cell match, independent of the neighborhood. In fairness to the NSP1 model, a 5x5 window kernel size was used to mimic the regional effect of the spatial model and calculated a global Two-Way statistic by averaging the fuzzy metric for all the cells that are predicted to be deforested in each model. The average Two-Way similarity is 0.64 for the NSP1 and 0.79 for the SLS1 model, a sizable difference in location accuracy. Thus, even when accuracy measurements use fuzzy or neighborhood metrics, the allocation performance of the spatial model is superior to the non-spatial model.

### 5. Simulating the Impact of New Roads on Deforestation and on Carbon Emissions

In this section, I use the estimated parameters from NSP1 and SLS1 and consider how the reduction of transport costs brought by planned roads will impact the probability of deforestation and carbon emissions. The federal and regional governments have planned the construction of several road segments (Fig 1, traced lines in black) to support a dual mode of transportation based on the extensive hydrologic network and roads instead of relying solely on water transport, as is the case today. These roads are designed to provide interconnectivity between important watersheds, thereby cutting transportation time and costs of getting products to central markets. For example, the Mazan—San Antonio del Estrecho will connect the Napo and Putumayo watersheds, the Jenaro Herrera–Angamos will connect the Ucayali and Yavarí Rivers, and the Orellana–Huallaga stretch will connect the Ucayalli and Huallaga Rivers and Yavarí River at the frontier with Brazil [31]. The road segments on the northwest part of Loreto are intended to provide access to important oil concessions and to secure the frontiers with Ecuador [51]. The segment from Marsella—Intuto along the Tigre River to the already built Iquitos—Nauta road will link Iquitos to the national road network once the segments to the Department of Amazonas are completed.

The effect of building these new roads on deforestation is estimated by accounting for the change in the “distance to roads” and “distance to deforestation” variables. Specifically, a GIS layer with the new roads was merged with the current roads’ layer and new distance values were calculated. These new variables substituted for the original variables and were ‘plugged-into’ the latent variable equation, and new probabilities were calculated. Let TC* be a vector containing these two new variables. Then the new raster probabilities NSP1* and SLS1* can be defined as:

${NSP1}^{*}\equiv {\hat{prob}}_{i}=\Phi (\hat{\alpha }{TC}_{i}^{*}+x_{i}\hat{\beta })$ for the NSP1 model, and

${{SLS1}^{*}\equiv \hat{prob}}_{ij}=\Phi (\hat{\alpha }{TC}_{ij}^{*}+x_{ij}\hat{\beta }+\hat{\rho }\sum_{k=1}^{m}w_{jk}{\hat{\theta }}_{k})$ for the SLS1 model, where the $\hat{\alpha ,}\hat{\beta },\hat{\rho }$ are the estimated regression parameters presented in Table 3 and $\hat{\theta }$ is the estimated regional effect.

Once in possession of these new probability surfaces for NSP1* and SLS1*, I generated 1,000 stochastic predicted deforestation landscapes for each model. In each realization, the probability of each cell *i* was compared to a random number draw from a uniform distribution. If the estimated probability for a given pixel was larger than the random number, I considered the pixel to be deforested [52, 53]. Next, I spatially intersected each stochastic predicted deforestation raster with an aboveground carbon dataset obtained from [54] and calculate the total amount of predicted emissions assuming that all carbon in a deforested cell is released into the atmosphere.

**Fig 5:**
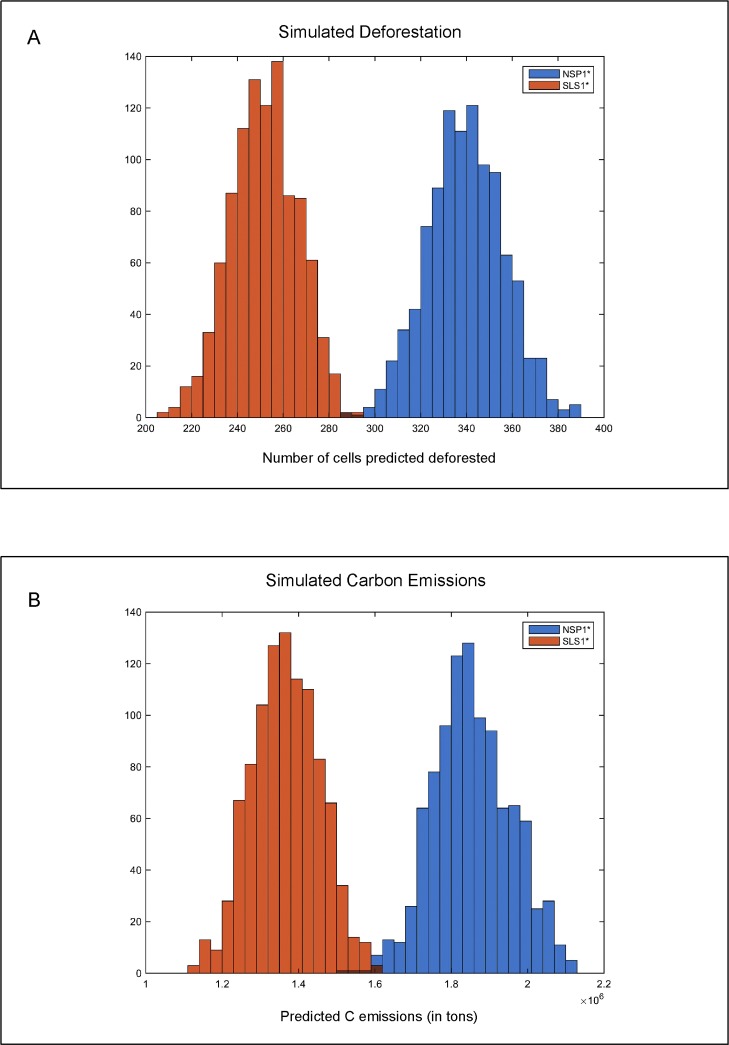
Predicted deforestation and carbon emissions. Histogram of cells predicted to be deforested from 1,000 stochastic simulations. (B) Associated carbon emissions from same simulations.

The average number of cells predicted to be deforested under the SLS1* model is 251 or only 203 km^2^ with a range between [205, 294] cells, whereas the NSP1* predicts a larger amount, 339 cells or 274 km^2^ with a range of [288,389] (Fig 5, panel A). This translates into average aboveground carbon emissions of 1.36 and 1.85 x10^6^ tons for the SLS1* and NSP1* models respectively (Fig 5 panel B). These differences in simulated deforestation among the spatial and non-spatial models highlights the ability of the former to differentiate cells with very low and cells with very high probabilities of deforestation whereas the NSP model generates a relatively more uniform (but still skewed to the left) distribution of probabilities with many cells showing mid-range probabilities. For instance, the SLS1* model generates 1,845 cells with probability greater than 0.5; at the same time 367,714 cells have probability less than 0.01. In contrast, only3 cells in the NSP1* have probability greater than 0.5 and only 244,754 cells with probability less than 0.01. Thus, the larger number of cells with probabilities in the mid-range increases their likelihood of being labeled ‘deforested’ in the stochastic analysis, leading to higher levels of predicted deforestation in the NSP1* model.

## 6. Discussion

This paper implements two sets of spatially explicit models, the non-spatial probit and the spatial probit with regional effects, to estimate the impact of new roads on deforestation in Loreto, Peru. It provides a policy relevant analysis for the western Amazon, while serving as an example of ways in which drivers of deforestation can be studied. The discussion below will address both policy implications of the analysis as well as computational issues related to the methods.

The methodological motivation for implementing the SLS model using raster data was twofold. First, spatially autocorrelated data generating processes are usually not incorporated into raster-based observations due to computational constraints. By adapting the SLS model to raster data, the computational requirements can be reduced significantly. Second, the SLS model replicates complex patterns of deforestation because the addition of the regional effect captures variations at a more local scale than the global parameters obtained in the non-spatial counterpart. There is however a ‘blocky’ or ‘pixelated’ aspect to the spatial deforestation pattern, a result of the strong regional effect captured by the estimated model that overpowers the variation of individual cells within each region. This pixelation could be reduced by making the region smaller. At the limit, if each observation is treated as a region, then the model will estimate spatial dependencies between individual pixels [10].

This region size reduction is attractive but it comes at the expense of computational cost. In the Loreto example, each region was defined as a 5x5 cell square window and spatial dependencies were assigned to occur between nearest neighbors. The 5x5 cell region size created 18,903 regions in total and the estimation procedure with 1,000 MCMC samplings took 7 hours and 5 min in a desktop computer equipped with Intel Pentium Xeon 3.20 GHZ processors and 24 GB of RAM memory. Attempts to use smaller regions were not possible due to insufficient RAM memory required to handle the neighborhood matrix and the log determinant calculation. Processing time usually cannot be reduced further because the MCMC procedure cannot be easily parallelized since each draw depends on the previous one. In principle, computational concerns aside, the method can be easily adapted to cases where cells fall within regions of different shapes or sizes (e.g. administrative boundaries such as census tracts, voting districts, etc.) or other neighborhood rules such as first or second order contiguity as long as the total number of regions is kept relatively small.

From a policy viewpoint, the results from both probit regressions show that deforestation is statistically correlated with transportation variables, but the marginal effects are small and translate into a modest simulated impact on deforestation, estimated to be between ~200–300 km^2^. This finding is contrary to the literature on infrastructure development in tropical regions, particularly for neighboring Brazil, where roads have been identified as one of the most important proximate causes of deforestation [5, 42]. This small effect is likely due to the current low levels of deforestation in Loreto that amount to less than 5% of the department. Large scale commercial agriculture is nonexistent and therefore there is currently little demand for large tracts of land for activities such as cattle ranching and soybean farming, as presently found in the Brazilian Amazon [12, 55].

Technological progress, introduction of new crops, and the dispossession of indigenous people’s land rights can of course change such a benign scenario, and the recent introduction of palm oil plantations serves as a warning that the analysis could be underestimating the impact of road construction in the region. Statistical land change models capture the relationship between *observed* outcomes and explanatory variables, and therefore reflect only past conditions. This means that projections of deforestation into the future assume that the processes driving deforestation are stationary and uniform over time [56].

Despite this caveat, both models, and the spatial one in particular, can still be useful in determining where deforestation is likely to occur, in addition to predicting the amount of deforestation. The results suggest that spatial probits using raster data can be useful to land change scientists and ecologists and also to decision makers as predictors of not only the amount of deforestation but more importantly of emergent fragmentation patterns [57]. This is of particular importance given fragmentation patterns play a key role in the viability of species’ survival, carbon storage, forest fire, and other ecosystem processes [58–60].

## Supporting Information

S1 File Distributions and parameters used in the Bayesian spatial probit model. (PDF)
