# Supplementary material for: A Spatial Probit Econometric Model of Land Change: The Case of Infrastructure Development in Western Amazonia, Peru
Source: PLoS One. 2016 Mar 24;11(3):e0152058. doi: 10.1371/journal.pone.0152058 (PMC4806871; doi:10.1371/journal.pone.0152058)
Supplement: S1 File — (PDF) [file pone.0152058.s001.pdf]

Supplementary Information S1 - Distributions and parameters used in the Bayesian spatial probit model estimation

Eugenio Y. Arima[1,*]

1 Department of Geography and the Environment, University of Texas, Austin, Texas, USA

¤305 E 23rd St Austin, TX 78712
*** arima@austin.utexas.edu**

SLS Spatial Probit Model

The model description presented here is heavily based on Smith and LeSage [1]. Following the notation described in the main manuscript, the spatial probit model can be written in full matrix notation as:

$$
\begin{aligned}
y^* &= X\beta + \Delta\theta + \epsilon \\
\theta &= \rho W\theta + \mu \\
\Delta &= \begin{pmatrix} \mathbf{1}_1 & & \\ & \ddots & \\ & & \mathbf{1}_m \end{pmatrix}
\end{aligned}
$$

where y^* is an $n \times 1$ latent variable vector, X is $n \times k$ matrix of explanatory variables, β is $k \times 1$, ϵ is $n \times 1$, and θ, μ are $m \times 1$. The matrix Δ assigns the same effect parameter to each observation within a region; $\mathbf{1}_j, j = 1, \ldots, m$ denotes an $(n_j \times 1)$ vector of ones where n_j is the number of observations within region j. The model assumes that $\mu \sim N(0, \sigma_\mu^2 \mathbf{I}_m)$ and $\epsilon|\theta \sim N(0, V)$, where $V = \begin{pmatrix} \nu_1 \mathbf{I}_{n_1} & & \\ & \ddots & \\ & & \nu_m \mathbf{I}_{n_m} \end{pmatrix}$

The matrix V allocates heterogeneity across regions through a set of variance scalars $\nu_j, j = 1, \ldots, m$ multiplied by the identity matrix $\mathbf{I}_{n_j}$.

Define an indicator function $\delta(A) = 1$ for positive outcomes and $\delta(A) = 0$ otherwise. Then, probability of deforestation can be written as:

$$
\begin{aligned}
Pr(y_{ij} = 1|y_{ij}^*) &= \delta(y_{ij}^* > 0) \\
Pr(y_{ij} = 0|y_{ij}^*) &= \delta(y_{ij}^* \leq 0)
\end{aligned}
$$

If we denote Y the $n \times 1$ vector of binary outcomes, we can write $Pr(Y = y|y^*) = \prod_{j=1}^{m} \prod_{k=1}^{n_j} \left\{ \delta(y_{jk} = 1)\delta(y_{jk}^* > 0) + \delta(y_{jk} = 0)\delta(y_{jk}^* \leq 0) \right\}$.

The function *semip_g* from LeSage's spatial econometric Matlab library [2] estimates this model through a hierarchical Bayesian estimation procedure using a Markov Chain Monte Carlo (MCMC) sampling approach. The following prior distributions are used:

$$\beta \sim N(c, T)$$
$$\frac{r}{\nu_j} \sim ID\chi^2(r)$$
$$\frac{1}{\sigma^2} \sim \Gamma(\alpha, \tau)$$
$$\rho \sim U[\lambda_{min}^{-1}, \lambda_{max}^{-1}]$$

β is assigned a normal conjugate prior with default values set to $c = 0$, and $T = 1e^{12}$, making it essentially diffuse. The variances σ^2 are given inverse gamma priors with parameters $\alpha = \tau = 0$, also making them diffuse. The prior for each ν_j is the inverse chi-square distribution, with hyperparameter $r = 4$. Finally, a uniform prior is employed for ρ, with $\lambda_{min} = 0, \lambda_{max} = 1$. The corresponding densities are:

$$\pi(\beta) \propto \exp[-\frac{1}{2}(\beta - c)' T^{-1}(\beta - c)]$$
$$\pi(\sigma^2) \propto (\sigma^2)^{-(\alpha+1)} \exp\left(-\frac{\tau}{\sigma^2}\right)$$
$$\pi(\rho) \propto 1$$

The implied prior density for the vector θ conditional on ρ, σ_μ^2, V is:

$$\pi(\theta|\rho, \sigma_\mu^2) \propto (\sigma_\mu^2)^{-\frac{m}{2}} |B| \exp\left(-\frac{1}{2\sigma_\mu^2}\theta' B' B \theta\right)$$
$$B = (I)_m - \rho W$$

The prior density ϵ given (θ, V) is

$$\pi(\epsilon|V) \sim |V|^{-1/2} \exp\left(-\frac{1}{2}\epsilon' V^{-1}\epsilon\right)$$

and the conditional prior of y^* given $(\beta, \sigma^2, \theta)$ is

$$\pi(y^*|\beta, \theta, V) \propto |V|^{-1/2} \exp\left\{-\frac{1}{2}(y^* - X\beta - \Delta\theta)' V^{-1}(y^* - X\beta - \Delta\theta)\right\}$$
$$= \prod_{j=1}^{m} \prod_{k=1}^{n_j} \left\{\nu_j^{-1/2} \exp\left[-\frac{1}{2\nu_j}(y_{jk}^* - x_{jk}'\beta - \theta_j)^2\right]\right\}$$

The posterior densities for $\beta, \theta, \rho, \sigma^2, \nu_j, y_{ij}^*$ can be found in the original manuscript by Smith and LeSage and estimation is achieved via MCMC, a method that samples sequentially from the complete set of posterior conditional densities for each one of the parameters, given initial guesses. This method produces a set of estimates that converge in the limit to the true joint posterior distribution of the parameters [3] .

References

1. Smith, T. E., and James P LeSage. A Bayesian Probit Model with Spatial Dependencies. In *Advances in Econometrics: Spatial and Spatiotemporal Econometrics*, edited by James P LeSage and Pace Kelley. 127-60. Oxford: Elsevier, 2004.

2. LeSage, J. Econometrics toolbox. Available: http://www.spatial-econometrics.com

3. Gelfand, A. E., and A. F. M. Smith. Sampling-Based Approaches to Calculating Marginal Densities. *Journal of the American Statistical Association* 85, no. 410 (1990): 398-409.
